# Global Gene Expression of Seed Coat Tissues Reveals a Potential Mechanism of Regulating Seed Size Formation in Castor Bean

**DOI:** 10.3390/ijms20061282

**Published:** 2019-03-14

**Authors:** Anmin Yu, Zaiqing Wang, Yang Zhang, Fei Li, Aizhong Liu

**Affiliations:** 1 Key Laboratory of Economic Plants and Biotechnology, Yunnan Key Laboratory for Wild Plant Resources, Kunming Institute of Botany, Chinese Academy of Sciences, Kunming 650201, China; yuanmin@mail.kib.ac.cn (A.Y.); wangzaiqing@mail.kib.ac.cn (Z.W.); lifeia@mail.kib.ac.cn (F.L.); 2 University of the Chinese Academy of Sciences, Beijing 100049, China; 3 Jiangxi Province Key Laboratory of Oil Crops Biology, Crops Research Institute of Jiangxi Academy of Agricultural Sciences, Nanchang 330200, China; pengzhang_123@163.com; 4 Key Laboratory for Forest Resources Conservation and Utilization in the Southwest Mountains of China, Ministry of Education, Southwest Forestry University, Kunming 650224, China

**Keywords:** castor bean, seed coat, seed size, lignin, cell division

## Abstract

The physiological and molecular basis of seed size formation is complex, and the development of seed coat (derived from integument cells) might be a critical factor that determines seed size formation for many endospermic seeds. Castor bean (*Ricinus communis* L.), a model system of studying seed biology, has large and persistent endosperm with a hard seed coat at maturity. Here, we investigated the potential molecular mechanisms underlying seed size formation in castor bean by comparing the difference between global gene expression within developing seed coat tissues between the large-seed ZB107 and small-seed ZB306. First, we observed the cell size of seed coat and concluded that the large seed coat area of ZB107 resulted from more cell numbers (rather than cell size). Furthermore, we found that the lignin proportion of seed coat was higher in ZB306. An investigation into global gene expression of developing seed coat tissues revealed that 815 genes were up-regulated and 813 were down-regulated in ZB306 relative to ZB107. Interestingly, we found that many genes involved in regulating cell division were up-regulated in ZB107, whereas many genes involved in regulating lignin biosynthesis (including several NAC members, as well as MYB46/83 and MYB58/63) and in mediating programmed cell death (such as CysEP1 and βVPE) were up-regulated in ZB306. Furthermore, the expression patterns of the genes mentioned above indicated that the lignification of seed coat tissues was enhanced and occurred earlier in the developing seeds of ZB306. Taken together, we tentatively proposed a potential scenario for explaining the molecular mechanisms of seed coat governing seed size formation in castor bean by increasing the cell number and delaying the onset of lignification in seed coat tissues in large-seed ZB107. This study not only presents new information for possible modulation of seed coat related genes to improve castor seed yield, but also provides new insights into understanding the molecular basis of seed size formation in endospermic seeds with hard seed coat.

## 1. Introduction

Seeds contain three major storage components, proteins, and carbohydratesand oils, which are the important sources for staple foods, livestock feed, and biofuel [1]. In angiosperms, seed development initiates with a double fertilization event that generates two zygotic products, the diploid embryo and the triploid endosperm [2]. The embryo and endosperm are enclosed by a seed coat, which is derived after fertilization from maternally integuments [3]. The final seed size and weight are determined by the coordination of cell proliferation and cell elongation of the three distinct constituents of the seed [4]. In most eudicot seeds, the growing cotyledons have to adjust their shape and size to the physical restrictions imposed by the seed coat (testa), such as in oilseed rape, pea, and the model plant Arabidopsis [3,5,6]. For typical endospermic seeds in monocot and dicot species, the persistent endosperm constitutes the bulk of the mature seed, such as rice, maize, castor bean, and coffee [7,8,9]. In recent years, the molecular mechanisms underlying seed size control have been studied extensively, and several genes that determine seed size by influencing the growth of the embryo, endosperm, and seed coat have been identified. For example, the best-known gene, DA1, controls seed size by limiting the duration of cell proliferation; the *da1-1* mutation causes an increase in seed volume, seed mass, embryo size, and cotyledon areas [10]. By contrast, endosperm development is represented by HAIKU1 (IKU1), IKU2, and MINISEED3 (MINI3) in the IKU pathway; these three genes are responsible for the early endosperm growth and seed size [11]. In addition, endosperm growth is also crucial for rice grain size, as TGW6 directly controls grain length and weight by affecting the time of cellularization in the early phase of endosperm development [12]. Studies on integument cell development reveal that seed coats act as physical constraints on embryo and/or endosperm growth by setting an upper limit to final seed size [13,14]. For example, MNT/ARF2 is a repressor of seed size, and thus extra cell division in the integuments of *mnt* (*megaintegumenta*) mutation leads to the formation of enlarged seed coats [15]. Therefore, final seed size is determined by the co-ordinated growth of three seed components, embryo, endosperm, and seed coat.

Castor bean (*Ricinus communis* L.) is one of the non-edible oilseed crops for biodiesel production, whose seed oil contain a high amount of unusual hydroxylated fatty acid ricinoleic acid [16]. Due to the rapid increase industrial demand for castor oil, there is an immediate requirement for enhanced castor seed yield through genetic crop improvement. As a representative of dicot endospermic seeds, castor bean possesses large and persistent endosperm, where the major storage reserves, including fatty acids, proteins, and starches accumulate [16]. In castor bean seeds, the embryos with two leafy cotyledons are embedded in the endosperm, and the mature seed coat consists of a hard-outer layer and a thin inner membrane [16,17]. Previous studies have been carried out to understand the genetic and epigenetic factors that affect seed size via the persistent endosperm in castor bean seeds [18,19,20]. The genetic and molecular mechanisms of seed size formation through control of seed coat have been discussed in many plants, such as Arabidopsis and the broad bean (*Vicia faba* L.) [3,21]. The color, shape, size, structure, and composition of seed coats vary significantly among different species. For example, the seed coats of Arabidopsis, rice, and maize are thin and tightly fused together with embryo or endosperm, while pine nut, broad bean, and castor bean are covered with hard and separable seed coats. The thickness and hardness of the seed coats are associated with the structure and chemical composition of the respective species [22]. Large amounts of cellulose, hemicellulose, and lignin are deposited in the secondary cell walls to reinforce the cell wall structure, making the outer surface of the seed coat impermeable to water, gases, or other physical environmental stresses during seed coat development [3]. Recently, the non-canonical C lignins were identified in the seed coat of castor bean, which were derived from caffeyl alcohols, similar to the precursors of classical G or S lignins, coniferyl or sinapyl alcohols, synthesized via the phenylpropanoid pathway [23]. Several enzymes involved in lignin synthesis have been identified, and the corresponding genes are well characterized, such as cinnamate 4-hydroxylase (C4H), 4-coumarate-CoA ligase (4CL), and O-methyltransferases [24,25]. In addition, the monolignol polymerization process is catalyzed by peroxidases (PRX) and laccases (LAC), and their roles in plant development have already been studied [26]. The expression level of LAC4 is regulated by miR397b, and determines lignin biosynthesis and seed yield in Arabidopsis [27]. Moreover, the biosynthesis of the secondary cell wall (including cellulose, hemicellulose and lignin) can also be activated by the secondary cell wall NAC transcription factors (VND6, VND7, SND1, etc.) as well as MYB transcription factors, i.e. MYB46 and MYB83 [28,29,30]. 

In this study, global differential gene expression analysis was carried out between the developing seed coat tissues of large-seed ZB107 and small-seed ZB306 using digital gene expression (DGE) technology. The results of cytological, chemical and transcriptional analysis revealed that seed coat plays an important role in seed size determination. This study not only presents new information for possible modulation of seed coat related genes to improve castor seed yield, but also provides novel insights into the molecular mechanisms of seed size formation in endospermic seeds with hard seed coat.

## 2. Results

### 2.1. Morphological Analysis of Seed Development and Determination of Seed Coat Fiber Compositions

**Figure 1:**
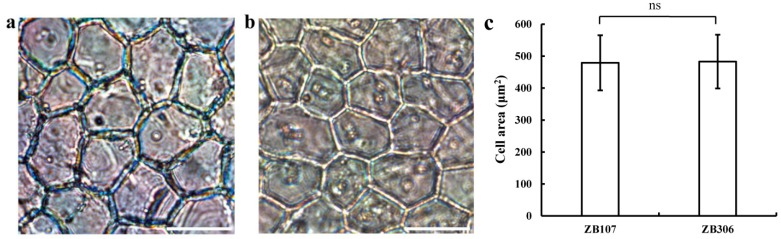
Seed coat morphology of the large-seed ZB107 and the small-seed ZB306. Light micrographs of the developing seed coat tissues dissected from the seed of ZB107 (**a**) and ZB306 (**b**) at the early stage. Scale bar =50 µm. (**c**) Comparison of cell size in seed coat tissues between ZB107 and ZB306. Values are reported as mean ± SD of at least ten independently seeds. ns, no significant difference (Student’s test).

We observed the seed development process (from pollination to seed maturity) of both large-seed ZB107 and small-seed ZB306 after artificial pollination, and found that the seed development of the two varieties underwent different durations, of 75 and 60 days, respectively. According to the change in embryo length and seed coat color [16], the process of castor bean seed development can be divided into three stages, i.e., the early stage, middle stage, and late stage. In the early stage, cell division in the embryo, endosperm, and seed coat tissues is active, but the enlargement in volume is slow; during the middle stage cell division and expansion is rapid with a fast storage material accumulation and a rapid increase in volume; and in the late stage, the volume and weight have reached the maximum and the lignification of seed coat begins, and the seed coat turns brown. Early, middle, and late stages in this study were recorded at 1–15, 16–30, 31–60 days, after pollination (DAP) in the small-seed ZB306, and at about 1–25, 26–45, and 46–75 DAPs in the large-seed ZB107, respectively. To determine whether the cell size or cell number contributes to the seed coat area between ZB306 and ZB107, we examined the cell performance of seed coat during seed development under the microscope. As shown in Figure 1, the cell size of seed coat tissues displayed no significant differences between ZB306 (at the 7 and 10 DAP) and ZB107 (at the 15 and 20 DAP) (see Figure 1). This observation suggests that the difference in seed coat area between ZB107 and ZB306 might be determined by cell number rather than cell size. In addition, the large-seed ZB107 underwent a longer development duration than the small-seed ZB306, implying that the seed coat tissues of ZB107 had a longer cell division phase and generated more cell numbers than ZB306.

**Figure 2:**
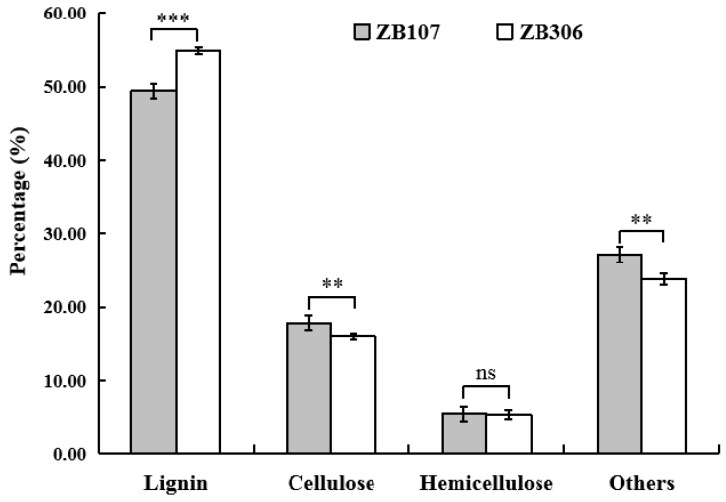
The fiber composition of seed coat ZB107 and ZB306.Values are reported as mean ± SD (n = 3). ** *p* < 0.01, *** *p* < 0.001, ns, no significant difference (Student’s test).

Since the cell lignification of seed coat tissues physically limited the seed development in volume [31], we investigated the lignin content of seed coat tissues for the mature seeds of ZB107 and ZB306 when the lignification process had finished. When measuring the fiber composition of lignified seed coat tissues for both ZB107 and ZB306, we found that the lignin proportions were different between two varieties. As shown in Figure 2, the lignin proportion in the mature seed coat tissues of ZB306 was significantly higher (54.80%) than that of ZB107 (49.48%), whereas the cellulose proportion in ZB306 was slightly lower (16.07%) than that of ZB107 (17.86%). A previous study has revealed that a higher proportion of lignin usually increased the hardness of the cell wall in seed coat tissues [32]. This observation suggests that the degree of seed coat cell lignification is lower in ZB107 compared with ZB306. 

### 2.2. Identification of Seed Coat Specially Expressed Genes and Global Analysis of the Differentially Expressed Genes in Seed Coat between ZB107 and ZB306

We performed the digital gene expression (DGE) profile of the seed coats ZB107 (at about 45 DAP) and ZB306 (at about 30 DAP), when seed volume nearly reached the final size and the lignification of seed coat had just started. In total, 7.2 million and 7.7 million reads were acquired from ZB107 and ZB306 (Table S1), of which 90.68% and 91.1% of the reads were mapped onto castor bean genome sequences [33]. The normalized expression level of each transcript was measured by reads per kb per million reads (RPKM), the low expression genes (0 ≤ RPKM < 1) accounted for 46.86% and 47.79%, whereas the high expression genes (RPKM > 60) only accounted for 10.67% and 10.88%, and the corresponding gene numbers were 3330 and 3397 in the seed coat of ZB107 and ZB306, respectively (Figure S1). These genes were used to identify genes specially expressed in seed coat by comparing these with the genes expressed in root and leaf using previous data [20]; the number of specially expressed genes in the seed coat of ZB107 and ZB306 was 29 and 45, respectively, and 49 genes were co-expressed in both ZB107 and ZB306 (Figure S2b). Furthermore, compared with the genes detected in embryo and endosperm [34], a significant fraction (17.1%) of genes was commonly present in three parts of castor seeds (in Figure S2a). The number of seed coat specific genes was 78 and 107 for ZB107 and ZB306, respectively, and 125 genes were co-expressed in both of ZB107 and ZB306. In addition, we performed the Kyoto Encyclopedia of Genes and Genomes (KEGG) enrichment analysis for genes specially expressed in seed coats, which were mainly involved in phenylpropanoid biosynthesis (ferulate-5-hydroxylase, 28140.m000099/28140.m000100), plant hormone signal transduction (xyloglucan endotransglucosylase/hydrolase protein 22 precursor, 30179.m000569), and transcription factors (27961.m000091, NAC domain-containing protein) (Figure S2c). 

**Figure 3:**
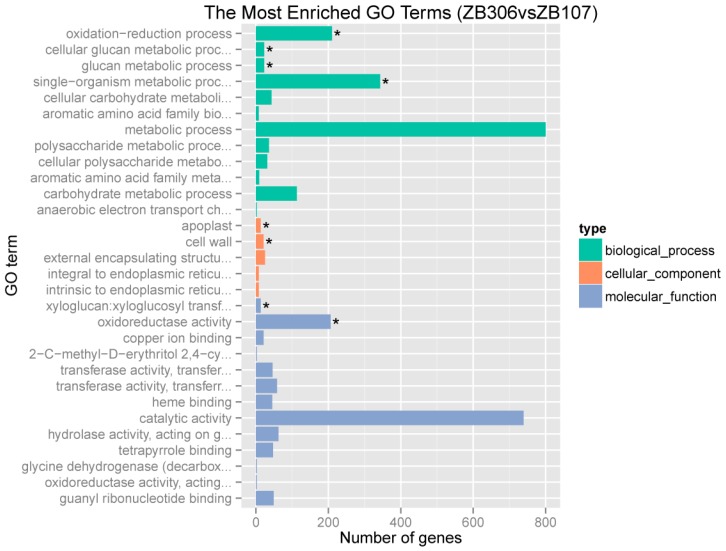
GO enriched analysis of the differentially expressed genes between ZB107 and ZB306. Significantly enriched GO terms are marked * (*p* < 0.05).

To investigate the transcriptional differences between the seed coat ZB107 and ZB306, we identified the differentially expressed genes using the statistical criteria of |log2 (fold change) | ≥ 1 and *p*-value ≤ 0.01. We found a total of 1628 differentially expressed genes between two varieties with 815 up-regulated genes and 813 down-regulated genes in ZB306 relative to ZB107. Gene Ontology (GO) functional enrichment analysis showed that the most enriched terms were glucan metabolic process (GO:0044042) and cellular glucan metabolic process (GO:0006073) in the biological process ontology category; cell wall (GO:0005618) in the cellular component; and xyloglucan: Xyloglucosyl transferase activity (GO:0016762) in molecular function (see Figure 3). Overall, these terms were related to the cell wall biosynthesis and the deposition of lignin in the secondary cell walls, which strongly suggested that the cell wall is an important factor affecting seed size through influencing the development of the seed coat.

### 2.3. Differential Expression of Lignin Biosynthesis Related Genes in Seed Coat between ZB107 and ZB306

As a three-dimensional polymer of phenylpropanoid units, monolignols are derived from the phenylpropanoid pathway, and then the polymerization of monolignols is performed by laccases and class III peroxidases [35]. Three main enzymes involved in the general phenylpropanoid pathway of successive deamination, hydroxylation, and esterification are: Phenylalanine ammonia-lyase (PAL, 30078.m002319, 28507.m000156), cinnamate 4-hydroxylase (C4H, 43540.m000048), and 4-coumarate-CoA ligase (4CL, 28429.m000109, 30131.m006921, 30073.m002251). Their expressions were up-regulated in ZB306 in the DEG profiles (Table 1). Moreover, cinnamoyl CoA reductase (CCR, 29588.m000854) and caffeoyl-CoA O-methyltransferase (CCoAOMT, 29968.m000634) in the monolignol-specific biosynthesis pathway were also up-regulated in ZB306. In addition, the final step of lignin biosynthesis was catalyzed by peroxidases and laccases, we only paid attention to those corresponding genes specially expressed in seed coat combined with the previous results. Peroxidase 10 (PRX10, 29676.m001629) and laccase 4 (LAC4, 29610.m000409) showed significantly higher expression in ZB306 as compared with ZB107. PRX72 (29634.m002067) and LAC5/17 (29751.m001786/30004.m000428) exhibited opposite expression patterns (Table 1). Thus far, it is still unknown which genes in the laccase and peroxidase gene families are involved in the formation of seed coat. We hypothesize that different laccase and peroxidase genes may play different roles in the oxidative polymerization of the monolignols during the formation of seed coats. Overall, the data reflects the differences of lignin biosynthesis in seed coat tissues of ZB107 and ZB306, which might be the special mechanism underlying seed size control in castor bean.

**Figure 4:**
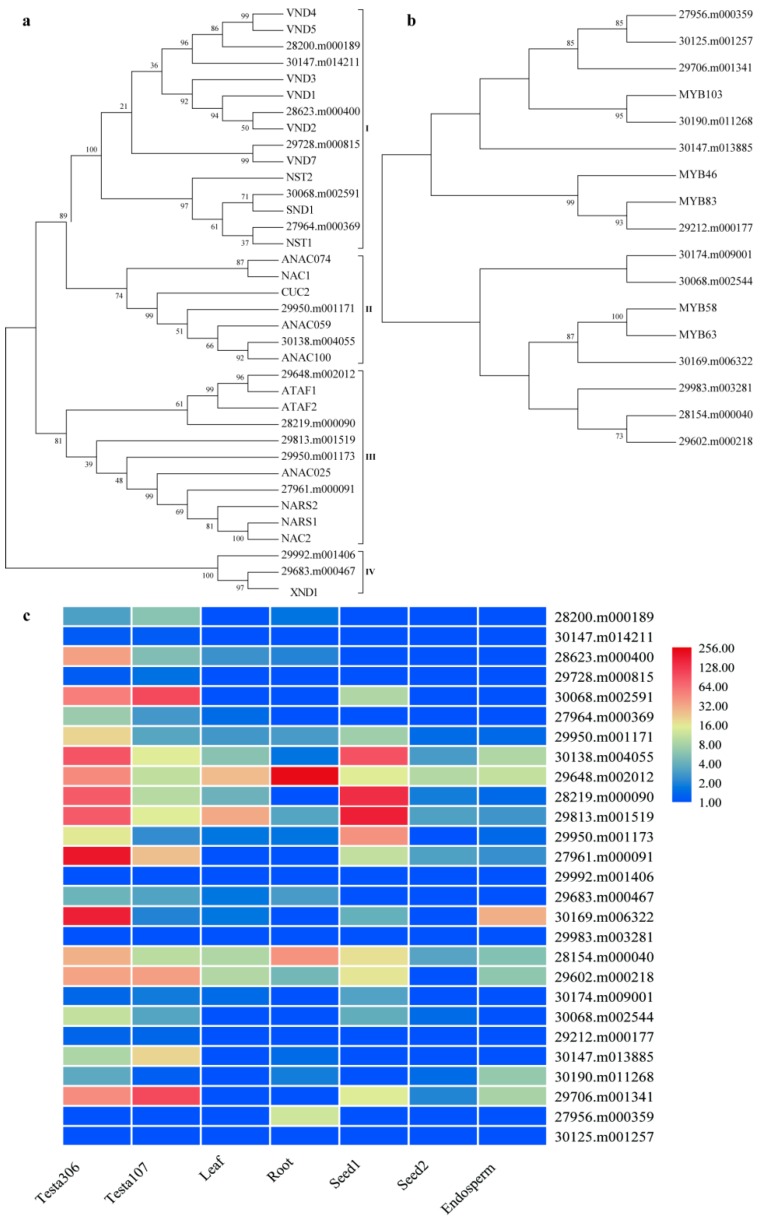
Phylogenetic and expression analyses of the NAC and MYB transcription factors related to secondary cell wall regulators. Phylogenetic analysis of NAC transcription factors (**a**) and MYB transcription factors (**b**) involved in the secondary cell wall biosynthesis. Bootstrap values are shown at nodes as a percentage from 1000 bootstrap replicates. (**c**) Gene expression analysis of castor bean NACs and MYBs related to secondary cell wall formation. The heatmap represents the relative TPM values of homologous genes in castor bean. The largest values are displayed as the reddest, the smallest values are displayed as the bluest. The labels of tissues and development stages are abbreviated using the following scheme: seed coat of ZB306 (Testa306) and ZB107 (Testa107) were collected at 35 DAP; leaf was collected from fully expanded young leaf, and root tips (root) were dissected, washed, and collected; seed 1 and seed 2 were collected at 15 DAP and 35 DAP; endosperm was dissected from the seeds at 40 DAP.

In addition, a group of secondary wall NAC (for NAM, ATAF1/2, and CUC2) domain transcription factors (TFs) and R2R3-MYB (R2R3 type of myeloblastosis domain protein) TFs are involved in the activation of the secondary wall biosynthetic program, consisting of cellulose, hemicellulose, and lignin. The secondary wall NACs act as the top-level master switches for secondary cell wall (SCW) formation, e.g., SND1 (for secondary wall-associated NAC domain protein), NST1 (NAC Secondary wall Thickening promoting factor 1), VND6 (Vascular-related NAC-domain 6), and VND7 [36]. We hypothesized that these NAC genes would activate the expression of several other TFs related to secondary wall thickening and lignin biosynthesis, such as MYB46 and MYB83 [37]. We identified a total of 15 and 12 genes in castor bean, which were homologous to NAC and MYB TF associated with SCW formation in Arabidopsis (Figure 4a,b). Furthermore, the expression patterns of these genes were evaluated in the seed coats of ZB107 and ZB306, as well as in other tissues (including leaf, root, seed 1, seed 2, and endosperm) which had been reported in the previous study [20]. Fifteen genes in the phylogenetic tree of secondary wall NAC TFs were classified into four subgroups (namely I to IV; Figure 4a). For six castor genes distributed in subgroup I, 28623.m000400 showed a closer relationship with VND2, and 29728.m000815 was closely related to VND7 (Figure 4a), while only the expression of 28623.m000400 was significantly up-regulated in the seed coat of ZB306, and the other five genes showed no significant difference in expression levels among various tissues (Figure 4c). In subgroup II, 30138.m004055 was closely related to ANAC100. In subgroup III, 29648.m002012 and 28219.m000090 were the orthologs of ATAF1 and ATAF2, while 27961.m000091 was closely related to NARS1/2. The expression of these genes in subgroup II and III was higher in the seed coat of ZB306 than ZB107, but 29648.m002012 in subgroup III showed preferential expression in root, as compared with other tissues. Furthermore, 29992.m001406 and 29683.m000467 belonged to subgroup IV, which were orthologous genes of the known Arabidopsis SCW regulator XND1, but their expressions were barely detectable in any of the tissues (Figure 4c). Likewise, all the MYB TFs related to secondary wall formation in castor bean could be classified into two subgroups with bootstrap support; each subgroup included 6 MYB genes from castor bean (Figure 4b). In subgroup I, 29212.m000177 and 30190.m011268 were closely related to MYB46/83 and MYB103, respectively. MYB46 and MYB83 were the direct downstream targets of secondary cell wall NACs in Arabidopsis, which could activate secondary cell wall deposition [38]. The protein structure of 30190.m011268 contained a typical R2R3 MYB domain, and the amino acid sequence shared 68% identity and 42% similarity with MYB46. MYB58 and MYB63 in subgroup II were the downstream targets of MYB46, and were transcriptional activators in the lignin biosynthetic pathway [36]. In subgroup II, 30169.m006322 was closely related to MYB58 and MYB63, and shared 46% and 48% similarities with MYB58 and MYB63, and the identities were 91% and 94% at the amino acid level. Moreover, 29212.m000177 and 30190.m011268 were expressed at very low levels, but 30169.m006322 in subgroup II showed a 7.38-fold up-regulation in the seed coat of ZB306 compared with ZB107 (Figure 4c). Taken together, these analyses identify the key TFs related to SCW, and suggest that the formation of seed coat was similar to vascular tissues, but not identical. These TFs may act as the master regulators of seed size via determining the lignin content in castor seed coats.

### 2.4. Differential Expression of Cell Development and PCD Related Genes in Seed Coats between ZB107 and ZB306

**Figure 5:**
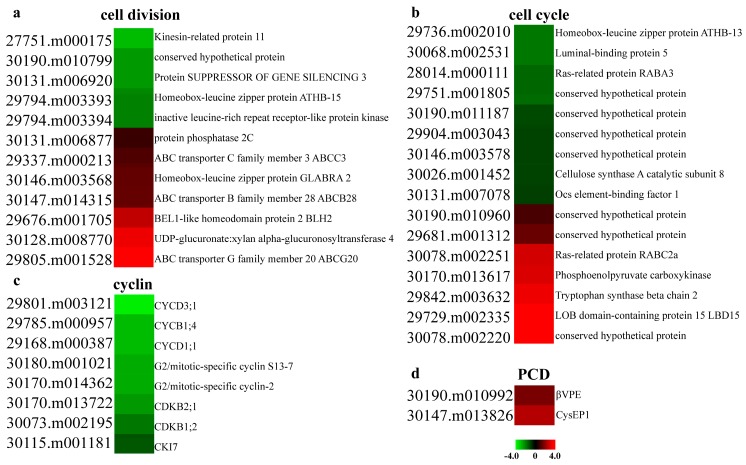
Heatmap comparison of the differentially expressed genes related to cell division (**a**), cell cycle (**b**), cyclin (**c**), and PCD (**d**) between ZB107 and ZB306. Genes are sorted in rising order according to fold change. The red and green colors represent up-regulated and down-regulated genes.

Seed coat development is the result of a series of overlapping events, including cell division, cell expansion, and cell differentiation [39]. Firstly, we investigated the expression patterns of cell division and cell cycle related genes, which are crucial for the regulation of cell development. While developing seeds at the end of middle stage were used, the process of cell division still took place among some cells in the seed coat tissues. Through GO enrichment analysis, we found that a total of 12 and 16 genes were linked to cell division and cell cycle, respectively. The process of cell division contained seven up-regulated genes and five down-regulated genes; BEL1-like homeodomain protein 2 (BLH2, 29676.m001705), ABC transporter G family member 20 (ABCG20, 29805.m001528), and ABC transporter B family member 28 (ABCB28, 30147.m014315) were up-regulated in ZB306 (Figure 5a). BLH2 had been designated as a negative regulator of cell division in Arabidopsis [40]. These genes involved in the cell cycle process contained seven up-regulated genes and nine down-regulated genes in ZB306, as compared with ZB107. For example, the expression of homeobox-leucine zipper protein ATHB-13 (29736.m002010) decreased 1.93-fold in ZB306. ATHB-13 had been determined as an important factor in the cell cycle, and the *athb13*-*1* loss-of-function lines exhibited shorter siliques, fewer seeds, and unfertilized ovules [41], suggesting that the reduced expression of ATHB-13 in ZB306 might explain why the seed size of ZB306 was smaller than ZB107. Moreover, eight cyclins and their associated kinases showed significantly down-regulated expression in ZB306 (Figure 5b), including one B type cyclin (CYCB1;4, 29785.m000957) and two D type cyclins (CYCD1;1/CYCD3;1, 29168.m000387/29801.m003121), which could control the cell number by regulating cell division [42]. Our results demonstrate that the expression of cell division related genes was higher in the seed coat of ZB107 compared with ZB306, meaning the number of seed coat cells was much higher in ZB107.

Next, we investigated the expression patterns of plant hormone signal transduction related genes. The expression levels of xyloglucan endotransglucosylase/hydrolase protein 22 precursor (XTH22 also known as TCH4, 30179.m000569) and CYCD3 (29801.m003121) in brassinosteroid (BR) signal pathway were higher in the seed coat of large-seed ZB107 (Table 2). However, two genes (28677.m000055 and 30131.m007029) encoding the nuclear growth repressor DELLA proteins in the gibberellin signaling pathway showed an opposite expression pattern. However, the expression level of 28677.m000055 was higher than 30131.m007029, and showed 1.05-fold up-regulation in ZB107. This suggests that the lower DELLA activity in ZB306 is an explanation for the reduced cell division rates. In addition, it has been reported that the exogenous application of gibberellic acid in table grape increased the deposition of lignin in the cortex [43]. These findings confirmed that gibberellic acid not only controlled cell division of the cortex, but also regulated the expression levels of genes related to lignin biosynthesis [44]. Taken together, our results indicate that plant hormones might play a useful role in cell division, cell expansion, and the lignification process.

During the phase of seed maturity, the outer integument became hardened and darkened, and the inner integuments compressed into a thin layer undergoing a series of programmed cell death (PCD) events in castor bean [45]. In a previous study, Rocha et al. identified two KDEL-tailed cysteine endopeptidases (CysEP1 and CysEP2) and two vacuolar processing enzymes (βVPE and γVPE) in the PCD process of castor seeds [46]. In DEG profiles, the expression levels of CysEP1 (30147.m013826) and βVPE (30190.m010992) were up-regulated in ZB306 (Figure 5d), while the expression of CysEP2 (29929.m004785) and γVPE (29794.m003351) could not be detected in the seed coat. The data suggests that, at the same seed coat developmental stages, the occurrence of PCD reach higher level in ZB306 than ZB107, which was similar to the expression pattern of lignin biosynthesis related genes. In sum, PCD was coupled with the lignification process, and the degree of these processes was stronger in small-seed ZB306 during seed coat development, but the underlying molecular mechanisms were poorly defined.

### 2.5. Experimental Validation of The Expression Patterns of Seed Coat Development-Related Genes

**Figure 6:**
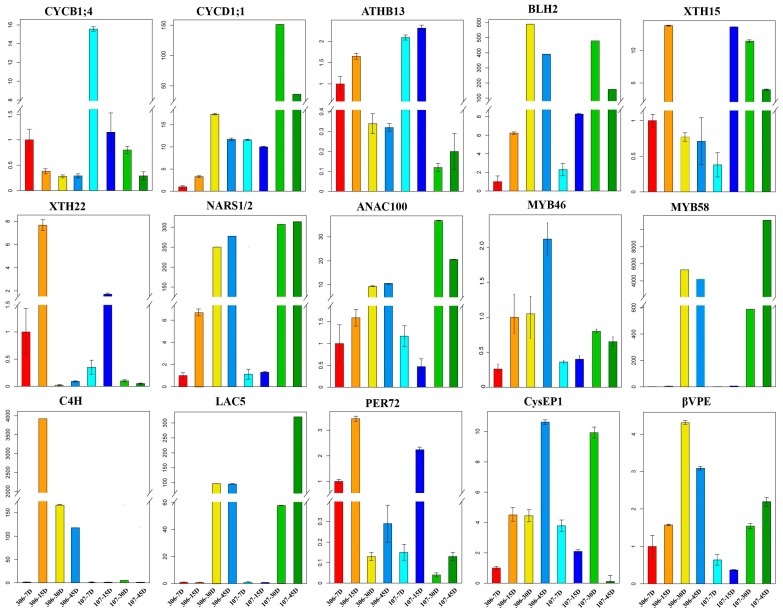
Relative expression levels of genes related to seed coat formation in ZB107 and ZB306. The x-axis represents the different stages of seed development at 7, 15, 30, and 40 days after pollination (DAP) for the seed coats of ZB306 and ZB107. The y-axis represents the gene expression level, which is the 2^−^^ΔΔCT^ value of qRT-PCR compared to the control gene. Data is shown as mean ± SD.

To further verify the differences in temporal and spatial expression of the genes related to cell growth, lignin biosynthesis, and PCD during seed coat development, we employed the quantitative real-time polymerase chain reaction (qRT-PCR) approach to detect the relative gene expression of each gene in the seed coat of ZB306 and ZB107 at different development stages. The results showed that expression levels of cell division related genes (e.g., CYCB1;4 and ATHB-13) in the seed coat of ZB107 were significantly higher than ZB306 at the same stages, with the exception of the cell division suppressor BLH2 (29676.m001705) (Figure 6). The expression level of CYCB1;4 (29785.m000957) was the highest at 7 DAP, and then gradually decreased with the subsequent development of the seeds ZB306 and ZB107. However, the expressions of CYCB1;4 (29785.m000957) and CYCD1;1 (29168.m000387) were significantly higher at 30 DAP in ZB107 than ZB306, this indicated that the process of cell division might last a longer time and contribute to the higher quantity of cells in the seed coat tissues of ZB107. Whereas, the expression of genes related to cell expansion, XTH15 (29993.m001055) and XTH22 (30179.m000569), were lower in ZB107 than ZB306 at 7 and 15 DAP, they showed opposite expression trends at 30 and 45 DAP. The results demonstrate that cell division and expansion mainly occur in the early stage of seed development, and that associated gene expression levels were higher in large-seed ZB107 than small-seeds ZB306 until the middle stage, which suggests that the increase in the number of seed coat cells results from the higher activity and a longer cell division phase.

For the process of lignification, expression levels of MYB46 (29212.m000177), MYB58 (30169.m006322), NARS1/2 (27961.m000091), ANAC100 (30138.m004055), LAC5 (29751.m001786), CysEP1 (30147.m013826), and βVPE (30190.m010992) reached higher levels from 30 DAP to 45 DAP. Meanwhile, the expression of MYB46 (29212.m000177), NARS1/2 (27961.m000091), ANAC100 (30138.m004055), C4H (43540.m000048), PER72 (29634.m002067), CysEP1 (30147.m013826), and βVPE (30190.m010992) were lower in ZB107 than ZB306 at 15 DAP during the early stage. The qRT-PCR results were consistent with the DEG data sets, showing that both the extent of lignification and PCD were enhanced in the seed coat of ZB306. Furthermore, our study demonstrates that the lignification and PCD of seed coat tissues starts earlier in the developing seed of ZB306 relative to ZB107; cell division was suppressed or terminated due to lignin deposition making the cell walls rigid in the seed coat of ZB306. 

## 3. Discussion

Seed size varies widely both within and among plant species, and the molecular mechanisms of seed size and weight formation are complex, involving the interactions of genetic and developmental factors, as well as environmental effects [3]. In general, the formation of seed size and weight is directly determined by the cell number and cell size of the main seed tissues (such as cotyledon and endosperm) during the cell division and cell expansion processes, respectively [47]. In monocotyledonous endospermic seeds such as rice, the formation of grain size, and weight is largely determined by the development of endosperm and the spikelet hull. For example, the key genes GS5 and GW8 have been identified as critical regulators for controlling grain width and weight through regulating cell number during seed development; while the regulators GL7 and SPL13 determine rice grain size through the regulation of cell size [48,49,50,51]. In dicotyledonous non-endospermic seeds such as Arabidopsis, the formation of seed size and weight is usually determined by the development of cotyledon. For instance, the regulator DA1 and CYCB1;4 have been identified to be critical for controlling seed size and weight through regulating cell division and increasing the cell numbers during seed development [10,42]. As for the dicotyledonous endospermic seeds with a hard seed coat (such as castor bean), little is known about the potential molecular basis of seed size formation. According to our previous observations, we found the seed coat size (large or small) of castor bean had been well developed in spite of the fact that the embryo and endosperm aborted at the early stage of seed development. This observation strongly suggests that the seed size of castor bean in volume might be eventually determined by the seed coat development. Thus, our current study is focused on dissecting the potential molecular basis of controlling the development of seed coat size by comparing the expressional difference of global genes during seed coat development between the large and small-seed varieties.

Clearly, the formation of seed coat size was determined by cell number rather than cell size in the seed coat tissues of castor bean according to our histological observation. GO enrichment analysis revealed higher expression levels of cell division related genes in the seed coat of ZB107, e.g., CYCB1;4 (29785.m000957) and CYCD3;1 (29801.m003121). In addition, the expression of CYCB1;4 (29785.m000957) was highest at the early stage of seed development when cell division mainly occurred. It has been demonstrated that the over-expression of CYCB1;4 increased seed size by influencing the cell number in the outer integument of Arabidopsis [42]. The over-expression of CYCD3;1 in Arabidopsis stimulated cell division and increased the cell number by controlling the length of the mitotic window [52]. Furthermore, two genotypes of *Vicia faba* differing in seed size also demonstrated a prolonged phase of cell division in large seeds, resulting in more cells in the seed coat and cotyledons [21]. These results demonstrated that a higher activity and longer phase of cell division significantly increased total cell numbers in the seed coat, and a greater cell number in the seed coats contributed to the larger seed size of ZB107. 

Earlier studies have reported the crucial role of integument cell elongation in regulating seed size of Arabidopsis, such as TTG2 (TRANSPARENT TESTA GLABRA2) and AP2 (APETALA2) [53,54]. In addition, TTG2 was involved in protoanthocyanidins and mucilage biosynthesis in the seed coat [54]. In castor bean, the seed coat specially expressed genes were mainly involved in phenylpropanoid biosynthesis and plant hormone signal transduction. TCH4 or BRU1 encoding XET (xyloglucan endotransglycosylase) proteins in BR signaling pathway, namely XTH22 (30179.m000569), and its expression level was higher in large-seed ZB107 at the middle stage. XTH22 played an important role in modifying xyloglucan polymers during cell wall loosening, which mediates cell expansion [55]. In previous studies, XTH22 showed a reduced expression in the BR receptor *bri1* mutants, suggesting that BR might control cell expansion by changing the extensibility of cell walls [56]. Altogether, cell expansion mainly occurred in the early and middle stages, and the increased number of cells resulted in higher cell expansion activity in large-seed ZB107, which might explain its larger seed coat area. As the seed coat of different species vary greatly in structure and composition, therefore, the molecular networks controlling seed size via seed coat may be different.

Furthermore, the castor seed coat was tough and hard, and nearly 50% of the fiber composition was lignin in the seed coat, suggesting a differential transcriptional regulatory network between castor bean and Arabidopsis. Lignin is an indispensable constituent of secondary cell walls, which is important for plant normal growth and seed coat formation [57]. The expression levels of these genes and TFs involved in lignin biosynthesis were higher in the seed coat ZB306, such as PAL (30078.m002319/28507.m000156), C4H (43540.m000048), NARS1/2 (27961.m000091), and MYB58 (30169.m006322), which lead to a higher lignin concentration in small-seed ZB306. It is well known that the inhibition of the expression of PAL, C4H, and 4CL significantly decreased lignin content in Arabidopsis and *Populus* [58,59]. Similarly, peroxidases and laccases involved in the monolignol polymerization also have been shown to affect lignin accumulation. In Arabidopsis, AtPRX64 influenced the lignification of root endodermis, and the mutation of AtPRX72 exhibited a reduction of lignin accumulation and stem height [26,60]. Interestingly, two types of laccase genes, LAC4 (29610.m000409) and LAC5/17 (29751.m001786/30004.m000428) showed opposite expression patterns between ZB107 and ZB306 (Table 1). While in the process of seed coat formation in Arabidopsis, only LAC15 was related to the lignin synthesis in seed coat cells [61], but this gene could not be detected in our study. It may be that members of the same gene family in different species undergo different regulations. In addition, the expressions of lignin related genes started to up-regulate earlier in ZB306 than ZB107, indicating that lignin deposition in the seed coat tissues occurred earlier in ZB306, as compared with ZB107, and that rigid secondary cell walls suppressed cell division. Altogether, the lignification process occurred earlier and the degree was higher in the seed coat tissues of ZB306, while cell division was arrested and the cell number decreased, leading to smaller seed coat area and seed size.

Similarly, PCD is an essential process in seed development and germination, such as PCD in the maternal tissues (pericarp, seed coat), nucellus, and endosperm [62,63,64]. The PCD related genes, CysEP1 (30147.m013826) and βVPE (30190.m010992), were highly expressed at the late stage when cell growth finished and a large amount of lignin had been deposited. In addition, the relative expression levels of CysEP1 and βVPE were higher in ZB306 than ZB107 at the early stage, and their expression patterns were similar to MYB46 (29212.m000177), MYB58 (30169.m006322), NARS1/2 (27961.m000091), and ANAC100 (30138.m004055). The NARS1/2 could control seed shape and size in Arabidopsis by regulating cell wall modification and PCD in the ovule integuments [65]. Likewise, the processes of lignification and PCD were tightly coupled during the xylem differentiation in Arabidopsis and the formation of fiber cells in *Populus* [66,67]. Overall, seed coat tissues should go through dramatic lignification and PCD processes to form the hard-outer seed coats and the shrinkage inner seed coats in castor bean. The onset of PCD together with lignification occurred earlier in the seed coat tissues of ZB306, and cell division was terminated prematurely.

**Figure 7:**
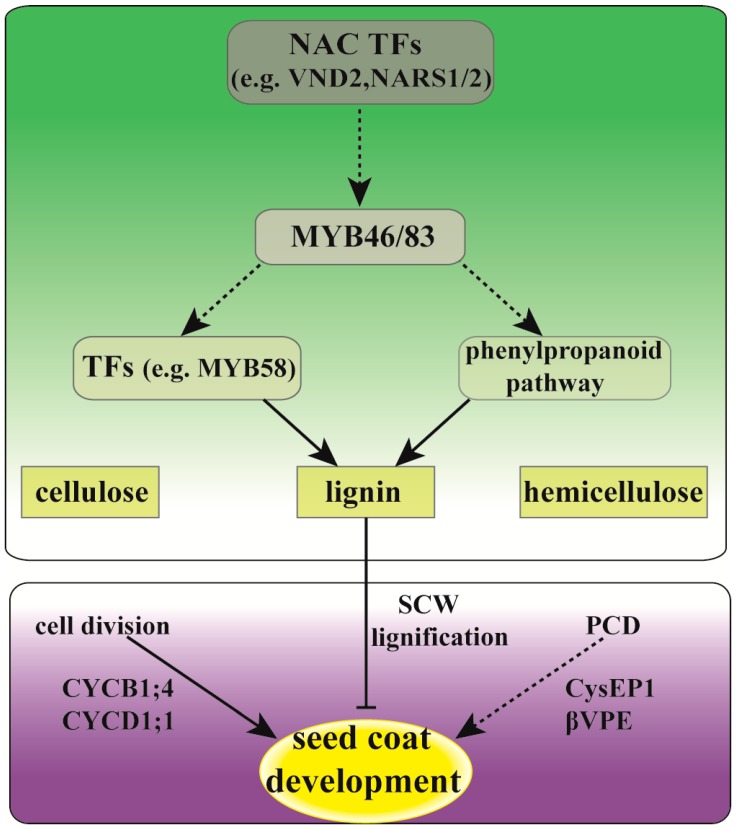
The hypothetical model of seed coat controlling seed size in castor bean. The seed coat cells undergo a period of cell division, which is in the control of CYCB1;4, CYCD1;1, and other cell division-related genes. Then, cell differentiation starts with the deposition of lignin in SCW, accompanied by the process of PCD. The NAC TFs (e.g., VND2, NARS1/2, ANAC100) acted as a master switch for the process of lignification, which could activate the expression of MYB46/83 [37]. Subsequently, the MYB58/63 TFs(which can directly regulate the biosynthesis of lignin) or the genes in the phenylpropanoid pathway were activated, and the lignin biosynthesis began.

In short, this study demonstrates that cell division and SCW lignification of seed coat cells might be critical to contribute to the cell number (enlargement of seed coat area) and deferring the lignification process of seed coat cells (prolonging the time of cell growth in large-seed coat). As shown in Figure 7, we proposed a hypothetical pathway to elucidate the potential regulation network of seed size formation in castor bean endospermic seeds. Firstly, we thought that seed size (in volume) formation was determined by the development of seed coat tissue. The period of cell division and expansion in seed coat tissues at the early and middle stages of seed development (i.e., the steady and fast enlargement of developing seeds in volume) might undergo a similar development process between the large and small-seeds, but the duration of cell division was longer in large-seed ZB107. Secondly, the differentiation of seed coats might start with the formation of the lignified cell wall, accompanied by cell cycle exit. The process of cell wall lignification occurs earlier in the small-seed ZB306 than that in the large-seed ZB107, resulting from the expressional up-regulation of NAC TFs (such as NARS1/2 and ANAC100) in ZB306 at the early stage, which are responsible for regulating the biosynthesis of lignin through activating the expression of downstream MYB46/83 [37]. Subsequently, the MYB58/63 TFs (which can directly regulate the biosynthesis of lignin) or the genes in the phenylpropanoid pathway were activated, and the lignin biosynthesis began. The lignification of seed coat tissues was advanced, and the phase of cell division was suppressed, resulting in a reduction of cell number and seed coat area in ZB306. In summary, based on our histological observation and global gene expressional differentiation within seed coat tissues, we investigated the potential molecular mechanisms underlying the formation of seed size between two phenotypes of castor bean differing in seed size, and concluded that a prolonged phase of cell division and the deferred lignification process of seed coat cells was the main cause of the formation of large castor bean seeds. This study might provide new insight into understanding the potential molecular mechanisms of seed size formation for plants that are endospermic and have a hard and lignified seed coat. Elucidating the genetic control of seed size could substantially accelerate crop improvement, which are important determinants of seed yield potential in castor bean.

## 4. Materials and Methods 

### 4.1. Plant Materials and Growth Conditions

Two castor bean varieties with contrasting phenotype for seed size and weight, large-seed ZB107, and small-seed ZB306, were used in this study. The castor plants were grown in the botanical garden, Kunming Institute of Botany at the Chinese Academy of Sciences, Yunnan Province, China, during the growing season under natural climate conditions. Individual flower buds were tagged at the first day after pollination (DAP). At least 10 seeds of ZB306 at 7 and 10 DAP, and corresponding to ZB107 at 15 and 20 DAP were collected for preparation of the histological samples. The seeds of ZB306 at 30 DAP and ZB107 at 45 DAP were used for DGE sampling, when the seed size reached a plateau and the lignification of seed coat had just begun. For the material samples of qRT-PCR analysis, the seeds of ZB306 were harvested at 7 and 15, corresponding to 15 and 25 DAP in ZB107, representing the early stage, while 30 and 45 DAP in ZB306, corresponding to 45 and 60 DAP in ZB107, were chosen to represent the middle and late stages, respectively. For easily understanding, we used the period of small-seed ZB306 to represent the corresponding developmental period in ZB107 in this study. For all seeds, except the histological samples, the outer seed coats were dissected from the fresh seeds, and flash frozen in liquid nitrogen. All dissected samples were stored at −80 °C for RNA extraction.

### 4.2. Histological Analysis

To measure seed coat cell size and number, the outer layer of the seed coats were freshly dissected from the seeds of ZB107 and ZB306, and soaked in ddH_2_O in Petri dishes (60 × 15 mm). The samples were cut into small pieces (5 × 5 mm) and mounted on glass slides before examination by Leica microscope (DM5500B, Bensheim, Germany). Twenty to forty images from independent sections per seed were photographed with Leica Microsystems (DFC450C). The cell area and cell number of the seed coat were measured using Image J software.

### 4.3. Determination of Seed Coat Fiber Composition and Contents

Seed coats were dissected from the mature dry seeds of ZB107 and ZB306, and heated at 65 °C in an oven until the weights did not change. Then, the dried samples were ground to powders, and 0.5 g material of each sample was placed in a filter-bag. First, the neutral detergent fibre (NDF) procedure of ANKOM 2000i Automatic fiber analyzer (ANKOM, USA) was used by adding 1 L neutral clean solution, 20 g sodium sulfite and 4 mL α-amylase in the material. Then, the residual material was taken out and soaked in acetone for 3–5 min and heated at 102 °C for 3 h in an oven, this step for residual material would be repeated later steps. Next, the contents of NDF, including hemicellulose, cellulose and lignin, were determined. The samples in the filter-bags were treated with 1 L acid detergent (20 g cetyltrimethyl ammonium bromide and 1 L 1 N sulphuric acid solution) for the hemicellulose to leave the residual material. At the end, the same operation was repeated with residual material. Then, the content of acid detergent fiber (ADF), including cellulose and lignin, were measured. The hemicellulose content was the NDF minus ADF content. Finally, samples in each bag were soaked in 72% (v/v) sulphuric acid solution for 3 h, then washed with water until pH became neutral. The the same operation was repeated with the residual material, and the content of acid detergent lignin (ADL) was estimated. ADL represented the whole lignin content, and the content of cellulose was the content of ADF minus ADL content.

### 4.4. RNA Extraction, Cdna Preparation and Illumina Sequencing

Total RNA from the seed coats of ZB107 and ZB306 were extracted using RNAprep pure Tissue Kit (Tiangen, Beijing, China) and the integrity and quality of the RNA was checked by 1% agarose gel electrophoresis and a NanoDrop 2000 spectrophotometer (Thermo Fisher Scientific, USA). The mRNA was purified from 3 μg total RNA using poly-T oligo-attached magnetic beads (Thermo Fisher Scientific, USA). Fragmentation of the mRNA was carried out and the cleaved RNA fragments were copied into first-strand cDNA using reverse transcriptase and random primers. Second-strand cDNA synthesis was subsequently performed using DNA Polymerase I and RNase H; double-stranded cDNA was purified and quantified. After size selection (150 to 200 bp), the adapter-modified DNA fragments were enriched by PCR. Next, library preparations were sequenced on an Illumina Hiseq 2000 platform according to the manufacture’s recommendations.

### 4.5. Analysis of DGE Data

Raw reads were initially processed to remove the adapter sequences, reads containing poly-N and low-quality reads. At the same time, Q20, Q30 and GC content were calculated. The high quality clean reads were mapped onto the castor bean reference genome (http://castorbean.jcvi.org/index.php) using Tophat [68]. HTSeq was used to count the reads mapped to each gene, in order to quantitate the genes expression level [69]. Then the read counts were normalized to reads per kilo base of exon model per million mapped reads (RPKM) using an in-house script. Differential expression analysis between ZB107 and ZB306 was performed using the DEGSeq R package [70]. A gene was considered significantly differentially expressed at the *p*-value ≤ 0.01 and |log2 (fold change) | ≥ 1.

### 4.6. Phylogenetic and Gene Expression Analysis

The amino acid sequences of NAC and MYB genes related to secondary cell wall formation from Arabidopsis and castor bean were retrieved in TAIR (https://www.arabidopsis.org/) and Castor Bean Genome Database (http://castorbean.jcvi.org/index.php). The sequence alignment was done using the Clustal W program, and phylogenetic analyses were performed with MEGA7.0 using the neighbor-join method with 1000 bootstrap generations [71]. The expression level of each gene in both the DGE data and the previous data from different tissues was normalized to the number of transcripts per million clean tags (TPM) [20]. Gene expression analysis was performed using the TBtools (https://github.com/CJ-Chen/TBtools).

### 4.7. qRT-PCR

Fourteen genes were selected to determine their expressions in seed coat at different periods of seed development. Total RNA was isolated and the quality was checked using the method described previously in Section 4.4, then first-strand cDNA was synthesized using TransScript All-in-One First-Strand cDNA Synthesis SuperMix for qPCR kit (TransGen Biotech, Beijing, China). All primers were designed using Primer 3 web (http://primer3.ut.ee/) and were listed in Table S2, and qRT-PCR was carried out using TransStart Tip Green qPCR SuperMix (TransGen Biotech, Beijing, China) with the Bio-Rad CFX96 system (California, USA). The cycling conditions were as follows: 2 min at 95 °C, followed by 40 cycles of 30 s at 95 °C, 30 s at 60 °C, and 30 s at 72 °C. A castor bean ACTIN2 gene was used as an internal reference and amplified in parallel with target genes, allowing gene expression normalization. The relative gene expression was presented using the 2^−ΔΔCT^ model [72]. Each PCR amplification was determined in at least three biological replicates and three technical replicates.

## Figures and Tables

**Table 1:** Identification of differentially expressed genes involved in lignin biosynthesis between ZB306 and ZB107 in seed coat.

Gene ^1^	Gene ID	ZB306 ^2^	ZB107 ^3^	Log2FC	*p*-Value	Gene Annotation
PAL	30078.m002319	596.57	44.30	3.75	1.1 × 10^−119^	Phenylalanine ammonia-lyase
PAL	28507.m000156	1307.44	73.76	4.15	3.5 × 10^−277^	Phenylalanine ammonia-lyase
C4H	43540.m000048	84.22	14.64	2.52	4.32 × 10^−13^	Trans-cinnamate 4-monooxygenase
4CL	28429.m000109	190.24	26.69	2.83	4.07 × 10^−31^	4-coumarate:CoA ligase 1
4CL	30131.m006921	2568.80	84.70	4.92	0	4-coumarate:CoA ligase 1
4CL	30073.m002251	122.55	20.20	2.60	6.14 × 10^−19^	4-coumarate:CoA ligase 2
CCR	29588.m000854	410.02	145.68	1.49	3.51 × 10^−29^	Cinnamoyl-CoA reductase 1
CCoAOMT	29968.m000634	202.55	71.36	1.51	2.19 × 10^−15^	caffeoyl-CoA O-methyltransferase
PRX10	29676.m001629	154.37	42.63	1.86	7.32 × 10^−16^	Peroxidase 10
PRX19	29900.m001566	0.00	27.62	−4.97	5.23 × 10^−08^	Peroxidase 19
PRX47	29983.m003295	3.87	27.99	−2.86	5.1 × 10^−06^	Peroxidase 47
PRX64	30170.m014275	0.00	52.27	−8.22	1.97 × 10^−12^	Peroxidase 64
PRX64	28962.m000432	7.21	34.10	−2.24	1.04 × 10^−05^	Peroxidase 64
PRX72	29634.m002067	154.73	2519.30	−4.03	0	Peroxidase 72
LAC4	29610.m000409	113.41	0.00	9.26	3.53 × 10^−22^	Laccase 4
LAC5	29751.m001786	37.27	131.03	−1.81	4.23 × 10^−14^	Laccase 5
LAC17	30004.m000428	80.18	1604.66	−4.32	0	Laccase 17

^1^ Abbreviations of gene name in this study. ^2^ ZB306: the RPKM values of ZB306. ^3^ ZB107: the RPKM values of ZB107.

**Table 2:** Identification of differentially expressed genes involved in plant hormone signal transduction pathway.

Pathway	Gene ^1^	Gene ID	ZB306 ^2^	ZB107 ^3^	Log2FC	Gene Annotation
ABA	PP2C	29739.m003582	38.33	78.40	−1.03	protein phosphatase 2c
ABA	ABF	29801.m003176	21.63	51.15	−1.24	DNA binding protein
Auxin	AUX|IAA	29598.m000460	48.70	114.35	−1.23	Auxin-responsive protein IAA16
Auxin	AUX|IAA	29841.m002748	3.87	59.12	−3.93	Auxin-induced protein AUX22
Auxin	AUX|IAA	29844.m003174	8.44	52.27	−2.63	Auxin-responsive protein IAA7
Auxin	AUX1	29908.m006146	5.45	26.13	−2.26	amino acid transporter
Auxin	AUX1	29969.m000264	3.52	25.21	−2.84	amino acid transporter
Auxin	GH3	30129.m000366	149.98	32.99	2.18	Indole-3-acetic acid-amido synthetase GH3.17
Auxin	SAUR	30131.m007151	6.51	26.50	−2.03	Indole-3-acetic acid-induced protein ARG7
Auxin	PIN3	29816.m000677	7.91	77.47	−3.29	Auxin efflux carrier component 3
BR	CYCD3	29801.m003121	4.04	48.37	−3.58	cyclin d
BR	TCH4	30179.m000569	0.00	241.12	−8.10	Xyloglucan endotransglucosylase/hydrolase protein 22 precursor
Cytokinine	A-ARR	28094.m000169	4.40	22.98	−2.39	two-component response regulator ARR-A
Cytokinine	A-ARR	29908.m006123	122.73	355.48	−1.53	Two-component response regulator ARR9
Ethylene	CTR1	29428.m000323	93.54	25.21	1.89	serine/threonine-protein kinase CTR1
Ethylene	ETR	29603.m000534	632.27	52.82	3.58	ethylene receptor
Ethylene	EBF1/2	28320.m001145	325.80	39.11	3.06	EIN3-binding F-box protein 1
Ethylene	EBF1/2	29848.m004629	258.29	64.31	2.01	EIN3-binding F-box protein 1
Ethylene	ERF1/2	29895.m000321	13.71	0.00	6.21	Ethylene-responsive transcription factor 1B
Ethylene	ETR	29986.m001660	111.30	45.04	1.31	ethylene receptor
GA	DELLA	28677.m000055	38.33	79.51	−1.05	DELLA protein GAI
GA	DELLA	30131.m007029	16.00	0.00	6.43	DELLA protein GAI
GA	GID1	29703.m001506	54.68	18.16	1.59	Gibberellin receptor GID1

^1^ Abbreviations of gene name in this study. ^2^ ZB306: the RPKM values of ZB306. ^3^ ZB107: the RPKM values of ZB107.

## Supplementary Materials

Supplementary materials can be found at https://www.mdpi.com/1422-0067/20/6/1282/s1. Table S1: Summary of DGE sequencing data and mapping results in the seed coat of ZB107 and ZB306, Table S2: Summary of primers used in this study, Figure S1: The distribution of RPKM values, Figure S2: Identification the seed coat specifically expressed genes in ZB107 and ZB306.

## Abbreviations

DGE	digital gene expression
PCD	programmed cell death
4CL	4-coumarate-CoA ligase
C4H	cinnamate 4-hydroxylase
PRX	peroxidases
LAC	laccases
DAP	day after pollination
PAL	phenylalanine ammonia-lyase
CCR	cinnamoyl CoA reductase
CCoAOMT	caffeoyl-CoA O-methyltransferase
TF	transcription factor
SCW	secondary cell wall
NST	NAC Secondary wall Thickening promoting factor
VND	Vascular-related NAC-domain
XTH	xyloglucan endotransglucosylase/hydrolase protein
BR	brassinosteroid
qRT-PCR	quantitative real-time polymerase chain reaction
NDF	neutral detergent fibre
ADF	acid detergent fiber
