# Supplementary material for: Global Gene Expression of Seed Coat Tissues Reveals a Potential Mechanism of Regulating Seed Size Formation in Castor Bean
Source: Int J Mol Sci. 2019 Mar 14;20(6):1282. doi: 10.3390/ijms20061282 (PMC6471003; doi:10.3390/ijms20061282)
Supplement: Supplementary file 1 [file ijms-20-01282-s001.pdf]

Table S1 Summary of DGE sequencing data and mapping results in the seed coat of ZB107 and ZB306

Sample	Total reads	Total mapped (Mapped Ratio)	Uniquely mapped (Mapped Ratio %)	Q20(%)	GC content (%)
ZB107	7,191,925	6,521,751 (90.68)	6,449,944 (89.68)	97.24	42.91
ZB306	7,665,568	6,983,662 (91.1)	6,878,496 (89.73)	97.17	42.91

Total mapped: reads mapped to the reference genome.

Uniquely mapped: reads mapped to the reference genome, excluding mismatching and empty positions.

Table S2 Summary of primers used in this study

Gene name	Primer name	Orientation	Primer sequence(5′-3′)
29785.m000957	CYCB1;4	Forward	CATGAATGTGCAGCCAGACA
		Reverse	TCCGGTGCCCATATCTCTTC
29168.m000387	CYCD1;1	Forward	CGATCCGTGACGCCATTTAG
		Reverse	GCTTAGCCCATCACACCATG
29736.m002010	ATHB13	Forward	TGATGCACTCCAAGCTCAGA
		Reverse	AGGCCTTGATGAAGAGCTGT
29676.m001705	BLH2	Forward	CCCCTAGCATCTCCTCATGG
		Reverse	CTTCTCTGGCATGTTTCCGG
29993.m001055	XTH15	Forward	AGTACCGGGTAACTCAGCTG
		Reverse	ATGGAGTAGGTGTGGAAGGC
30179.m000569	XTH22	Forward	TGGCTGCCTATCCTTGTCAA
		Reverse	AGAGTTGCCAGGTACAAGCT
29212.m000177	MYB46	Forward	AATCCTTCCACAGCATCACC
		Reverse	CCGGCCGTTATTAGTAAGCA
30169.m006322	MYB58	Forward	CCATTTGCCGGGTAGAACAG
		Reverse	TCACAGAGCCATGGTTCCAT
27961.m000091	NARS1/2	Forward	TAACAATCGGCAGCCAACAG
		Reverse	CTAGCAGCCATGTCAACGTC
30138.m004055	ANAC100	Forward	CCACAACCTCCCCAAAACTG
		Reverse	TAGGATTGGAGAAGCAGGGC
43540.m000048	C4H	Forward	GACCTGGCAACCAAATCACT
		Reverse	GCAGGAATGTCGTACCCACT
29751.m001786	LAC5	Forward	CTTTGTTGTTGGCCAAGGTT
		Reverse	AAACCCCAGCTTGTATGCAC
29634.m002067	PER72	Forward	AAGCATCCATGGAACTGGTC
		Reverse	ACCTCTCCCCTTGAACCTGT
30147.m013826	CysEP1	Forward	GCAAAGGGGTGGCATAACAA
		Reverse	AACCCACGATTGCTACTCCA
30190.m010992	βVPE	Forward	ACCAGGTCTCCTTGCTTCTC
		Reverse	ATAATAACCCCAGGCCTCGG

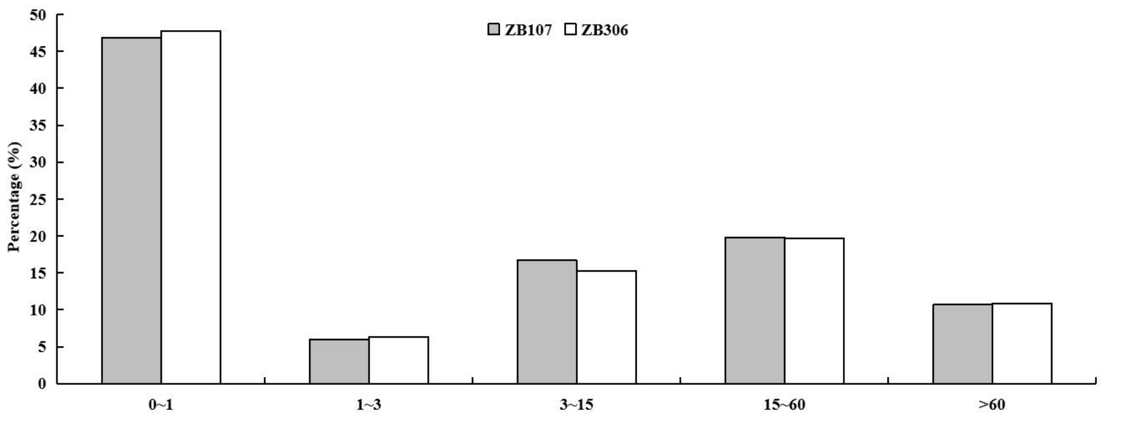

Figure S1 The distribution of RPKM values. The x-axis represents the different RPKM values. The y-axis represents the percentage of gene number.

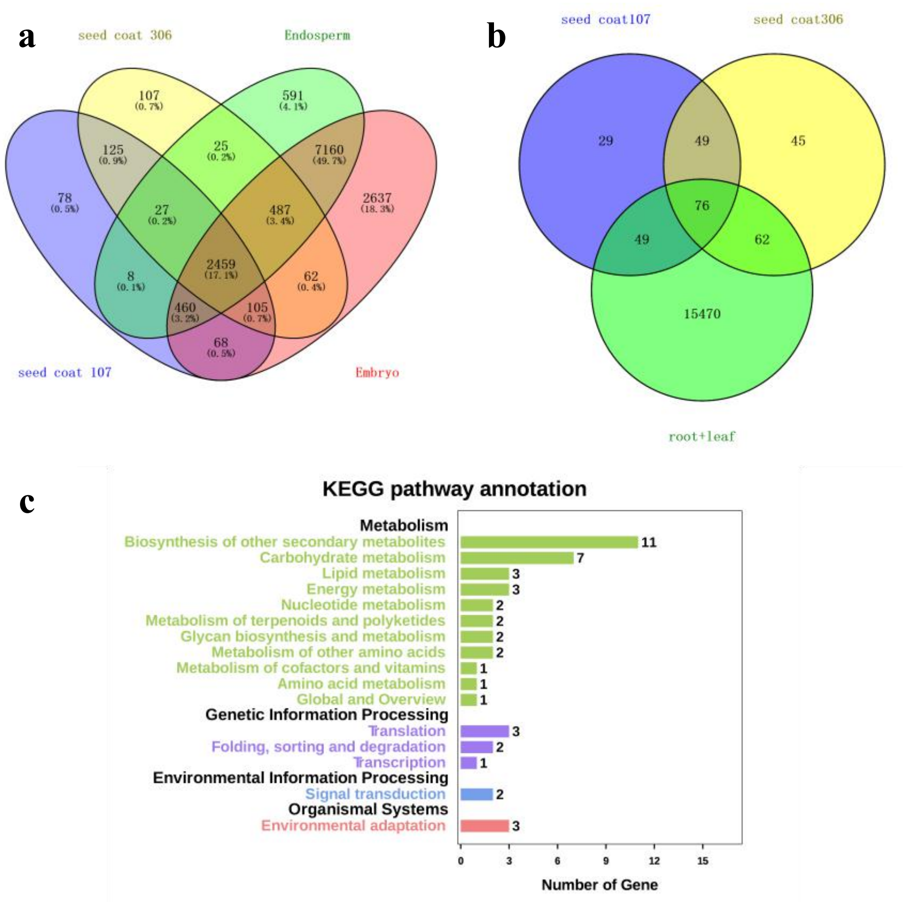

Figure S2 Identification the seed coat specifically expressed genes in ZB107 and ZB306. (a) Venn diagram showing the numbers of tissue specific genes among the seed coat of ZB306 (seed coat 306), seed coat of ZB107 (seed coat 107), endosperm and embryo of ZB306. (b) Venn diagram showing the numbers of tissue specific genes among the seed

coat of ZB306, seed coat of ZB107, root and leaf of ZB306. (c) KEGG pathway categories of the seed coat specifically expressed genes.
